# Is perioperative fasting associated with complications, length of hospital stay and mortality among gastric and colorectal cancer patients? A cohort study

**DOI:** 10.1590/1516-3180.2020.0084.R1.30062020

**Published:** 2020-10-09

**Authors:** Isabel Pinto Amorim das Virgens, Ana Lúcia Miranda de Carvalho, Yasmim Guerreiro Nagashima, Flavia Moraes Silva, Ana Paula Trussardi Fayh

**Affiliations:** I BSc. Dietitian and Master's student, Postgraduate Program on Health Sciences, Universidade Federal do Rio Grande do Norte (UFRN), Natal (RN), Brazil.; II MSc. Dietitian, Surgical Oncology Department, Luiz Antônio Hospital, Liga Norteriograndense Contra o Câncer, Natal (RN), Brazil.; III BSc. Dietitian, Surgical Oncology Department, Luiz Antônio Hospital, Liga Norteriograndense Contra o Câncer, Natal (RN), Brazil.; IV PhD. Dietitian and Adjunct Professor, Department of Nutrition, Universidade Federal de Ciências da Saúde de Porto Alegre (UFCSPA), Porto Alegre (RS), Brazil.; V PhD. Dietitian and Associated Professor, Department of Nutrition, Universidade Federal do Rio Grande do Norte (UFRN), Natal (RN), Brazil.

**Keywords:** Fasting, Perioperative care, Medical oncology, Enteral nutrition, Length of stay, Nutrition, Gastrointestinal cancer, Surgery, Early enteral feeding, Perioperative fasting

## Abstract

**BACKGROUND:**

During a surgical procedure, patients are often subjected to fasting for times that are more prolonged than the ideal, which may lead to complications.

**OBJECTIVE:**

To evaluate the duration of perioperative fasting and its association with postoperative complications, length of hospital stay (LOS) and mortality among gastric and colorectal cancer patients.

**DESIGN AND SETTING:**

Cohort study developed in a surgical oncology hospital in the city of Natal (Rio Grande do Norte, Brazil).

**METHODS:**

Patients aged over 18 years were included. The Clavien-Dindo surgical complication scale was used to evaluate occurrences of postoperative complications. LOS was defined as the number of days for which patients stayed in the hospital after surgery, or until the day of death.

**RESULTS:**

Seventy-seven patients participated (59.8 ± 11.8 years; 54.5% females; 70.1% with bowel tumor). The incidences of postoperative complications and death were 59.7% and 3.9%, respectively. The duration of perioperative fasting was 59.0 ± 21.4 hours, and it was higher among non-survivors and among patients with prolonged hospital stay (≥ 6 days). For each one-hour increase in the durations of perioperative and postoperative fasting, the odds of prolonged hospitalization increased by 12% (odds ratio, OR = 1.12; 95% confidence interval, CI 1.04-1.20) and 5% (OR = 1.05; 95% CI 1.02-1.08), respectively.

**CONCLUSION:**

Prolonged perioperative fasting, especially in the postoperative period, was observed in a sample of patients with gastric and colorectal cancer, and this was an independent predictor of LOS.

## INTRODUCTION

Gastrointestinal cancers, including cancers of the esophagus and stomach, colon and rectum, liver, gallbladder, pancreas, small intestine, appendix and anus, collectively represent one of the greatest public health issues, given that they lead to almost 4.5 million deaths worldwide per year.^1^ Although significant progress has been made around the world, towards reducing incidence and mortality rates through improving survival, gastrointestinal cancer is rarely detected early, and the prognosis remains poor.^2^

Several types of surgery are helpful to patients with cancer, and this can be used in combination with other types of treatment. The main goal of surgery to treat cancer is to completely remove the tumor or cancerous tissue from a specific place in the body (curative surgery), and it is most effective when performed at an early stage.^3^ Although nowadays surgery can be considered to be a minimally invasive procedure, it can lead to complications when performed on patients with altered nutritional status.^4^ Thus, perioperative care is extremely important for successful surgical treatment.

Unfortunately, before a surgical procedure, the real duration of fasting is often more prolonged than what is prescribed. This arises for a variety of reasons relating to the hospital routine. The routine of 12 hours or nocturnal fasting is the most common protocol before elective surgery, in order to reduce complications and adverse events relating to the gastrointestinal and respiratory tract, due to the anesthesia.^5^ Preoperative nocturnal fasting was instituted when anesthetic techniques were still quite rudimentary and chloroform was used. Its main objective was to avoid respiratory complications due to vomiting and aspiration of gastric contents.^6^ Nowadays, shortening of fasting through use of carbohydrate beverages, such that it is started between six and two hours before surgery, can bring benefits regarding glycemic and functional parameters.^6^ It also reduces hospitalization and does not increase the aspiration risk among patients undergoing elective surgery. Thus, shortening of fasting contributes towards maintenance of nutritional status.^7^

Despite the recommendations, implementation of these protocols is still only just beginning in many countries, including Brazil. So far, a shortened perioperative fasting period for surgical cancer patients has not yet been studied.

## OBJECTIVE

The objective of this study was to evaluate the duration of perioperative fasting and its association with postoperative complications, length of hospital stay (LOS) and mortality in a sample of surgical patients with gastric and colorectal cancer.

## METHODS

### Design, sample and ethics

This was a prospective cohort study conducted between December 2017 and December 2018, in a surgical oncology hospital in the city of Natal, RN, Brazil. The study protocol was approved by the Human Research Ethics Committee (under protocol number 2.315.013) on October 5, 2017. Gastric and colorectal cancer patients aged over 18 years who were scheduled to undergo open surgery procedures were included. The exclusion criterion was the presence of other diseases that cause a decrease in muscle mass, such as heart failure, acquired immunodeficiency syndrome, inflammatory bowel diseases, non-cancer liver diseases or tuberculosis. Patients undergoing palliative surgery (for whom only exploratory laparotomy and biopsy were performed) were also excluded because of their extensive disease verified during the surgery.

All subjects gave their written informed consent, in accordance with the Declaration of Helsinki.

### Procedures

All patients with gastric and colorectal cancer who were scheduled to undergo a surgical oncological procedure during the study period was invited to participate. Before the surgical procedure, data to characterize the sample were collected from the patients’ medical records and through in-person interviews: sex, age, ethnicity, presence of comorbidities (diabetes and/or hypertension) and smoking, along with information about the tumor, any neoadjuvant treatment involving chemotherapy and/or radiotherapy that had been undertaken, and the individual's functional capacity in terms of the Eastern Cooperative Oncology Group Performance Status (ECOG-PS).^8^ All patients were followed up for 30 days after the surgical procedure, regardless of the length of their hospital stay, or until the time of death. After the surgery, information about the duration and type of surgery performed, and about occurrences of postoperative complications, were collected.

Body mass index (BMI, kg/m^2^) was calculated from height and weight. From this, the patients were classified as underweight, normal weight, overweight or obese, in accordance with the World Health Organization (WHO) criteria.^9^ Nutritional status was also evaluated by means of the Patient-Generated Subjective Global Assessment (PG-SGA). Score A indicated a patient without malnutrition, while scores B and C represented malnourishment (suggestive of moderate malnutrition and severe malnutrition, respectively).^10^

Fasting was considered to consist of absence of oral (food and drink), enteral or parenteral nutrition. Patients were asked directly what the duration of their fasting before and after surgery had been and confirmation of this was sought from the electronic records, whenever possible. Only when it was not possible to obtain reliable information regarding the duration of fasting from the patient was information from the medical records used.

### Outcomes

The outcomes from this study comprised surgical complications, duration of hospitalization and incidence of hospital death. The Clavien-Dindo scale was used to evaluate surgical complications.^11^ This classifies complications in ascending degrees, from I to V, according to their severity, and in our study the version of the scale translated and adapted for use in Brazilian Portuguese was used.^12^ Based on other similar studies in the literature,^13^^–^^15^ only complications classified as grade II onwards were considered in the present study. Grade II complications include infectious processes treated with antibiotics, need for blood transfusion and parenteral nutrition. Complications of grades III, IV and V include surgical re-interventions for correction of fistulas, intra-abdominal abscess and evisceration, intensive care unit (ICU) hospitalizations for treatment of abdominal sepsis, and death. The LOS was defined as the number of days for which patients stayed at the hospital after surgery, or until the day of death.

### Statistical analysis

Descriptive statistics were calculated and the data were expressed as the mean and standard deviation for quantitative parametric variables; as the median and interquartile range (P25-P75) for quantitative nonparametric variables; or as the absolute and relative frequency for categorical variables. The normality of quantitative variables was assessed using the Kolmogorov-Smirnov test. Clinical and nutritional characteristics were compared between groups using the chi-square test for categorical variables and using the independent t test (parametric variables) or Mann-Whitney test (nonparametric variables) for continuous variables. Correlations between the duration of fasting and the length of hospital stay after surgery were evaluated using Spearman's coefficient.

Logistic regression was performed considering the length of hospital stay (categorized according to the median of six days), incidence of postoperative complications and death in hospital as dependent variables; and the duration of perioperative or postoperative fasting as independent variables. The covariates included in the adjusted model were the patients’ ages, nutritional status according to PG-SGA, tumor site and stage, duration of postoperative hospital stay and occurrence of complications in the postoperative period. The durations of perioperative or postoperative fasting were considered to be continuous variables in the model constructed. All the analyses were performed in the SPSS 20.0 software and P-values < 0.05 were considered statistically significant.

## RESULTS

A total of 140 patients were initially screened before surgery, but 63 of them were excluded because their data regarding the duration of pre or postoperative fasting were incomplete. Therefore, 77 patients of mean age 59.8 ± 11.8 years were enrolled in this study. Table 1 describes the baseline demographic characteristics of the sample.

**Table 1:** Clinical and nutritional features of the surgical patients (n = 77)

Variables		n	%
**Gender (%)**			
	Female	42	54.5
**Ethnicity (%)**			
	Non-Caucasian	58	75.3
**Hypertension (%)**			
	Yes	45	58.4
**Diabetic (%)**			
	Yes	17	22.1
**Smoking history (%)**			
	Yes	28	36.4
**Alcohol consumption (%)**			
	Yes	26	33.8
**Tumor site (%)**			
	Gastric	23	29.9
	Colorectal	54	70.1
**Clinical tumor stage**			
	I	17	22.1
	II	17	22.1
	III	22	28.6
	IV	14	18.2
	Unknown	7	9.1
**Neoadjuvant treatment**			
	Yes	29	37.7
**BMI classification**			
	Undernutrition	4	5.2
	Normal weight	31	40.3
	Overweight	32	41.6
	Obesity	10	13.0
**PG-SGA classification**			
	A	49	63.6
	B or C	28	36.4
**ECOG-PS**			
	**0**	36	46.8
	1	29	37.7
	2	9	11.7
	3	2	2.6
	4	1	1.3
**Complications**			
	Yes	46	59.7

BMI = body index mass; PG-SGA = Patient-Generated Subjective Global Assessment; ECOG-PS = Eastern Cooperative Oncology Group Performance Status.

The mean duration of preoperative fasting was 15.9 ± 5.3 hours, while the median duration of postoperative fasting was 39.9 (19.5-46.9) hours. The duration of perioperative fasting was 59.0 ± 21.4 hours. Death occurred in the case of three individuals, of whom two were colorectal cancer patients. Table 2 shows the patients’ clinical and nutritional features according to the cancer site. In general, gastric cancer patients had worse status performance and presented longer duration of surgery and more complications than did colorectal cancer patients. Also, LOS was greater among gastric cancer patients. Although the surgical complications differed between the groups, the time taken (in days) for complications to appear was not different.

**Table 2:** Comparison between clinical and nutritional characteristics according to cancer site

	Total (n = 77)	Gastric cancer (n = 23)	Colorectal cancer (n = 54)	P
Undernutrition^1^	28 (36.4%)	10 (43.5%)	18 (33.3%)	0.397*
Excess weight^2^	42 (54.5%)	13 (56.5%)	29 (53.7%)	0.820*
Good performance status^3^	65 (84.4%)	16 (69.6%)	49 (90.7%)	0.019*
Clinical tumor stages III and IV	39 (50.6%)	13 (56.5%)	23 (42.6%)	0.083*
With surgical complications	46 (59.7%)	18 (78.3%)	28 (51.9%)	0.031*
Preoperative fasting (h)	15.90 ± 5.31	15.29 ± 3.81	16.16 ± 5.85	0.514#
Postoperative fasting (h)	39.92 (19.54-46.96)	41.00 (19.08-63.33)	39.87 (16.62-46.50)	0.501π
Perioperative fasting (h)	58.95 ± 21.45	61.96 ± 26.91	57.67 ± 18.79	0.425#
Duration of surgery (min)	183.75 ± 68.36	215.4 ± 73.6	170.3 ± 61.9	0.007#
Length of hospital stay (days)	6.0 (4.0-8.0)	8.0 (7.0-12.0)	5.0 (4.0-7.0)	< 0.001π
Number of days taken for complications to appear	3.0 (2.0-8.0)	4.0 (1.8-7.0)	3.0 (2.0-11.0)	0.847π

1 According to the Patient-Generated Subjective Global Assessment (PG-SGA);

2 According to body mass index ≥ 25 kg/m^2^;

3 Eastern Cooperative Oncology Group Performance Status ≤ 1;

* P-value with chi-square test;

# P-value with independent t test

π P-value with Mann-Whitney test.

The duration of fasting did not differ between patients with and without complications in the postoperative period, as demonstrated in Table 3. Survivors had shorter postoperative and perioperative fasting, in comparison with non-survivors. Patients with longer hospitalization after surgery had longer durations of fasting during the postoperative and perioperative period than patients with hospital stays after surgery shorter than six days.

**Table 3:** Duration of perioperative fasting among patients with gastric and colorectal cancer according to survival, incidence of complications and length of hospital stay (LOS) after surgery

	Survivors (n = 74)	Non-survivors (n = 3)	P
Preoperative fasting (h)	15.9 ± 5.4	16.2 ± 5.2	0.956^1^
Postoperative fasting (h)	39.2 (19.1 - 46.7)	77.4 (16.9-65.5)	0.038^2^
Perioperative fasting (h)	57.9 ± 20.6	97.0 ± 21.2	0.010^1^
	**Without complications (n = 31)**	**With complications(n = 46)**	**P**
Preoperative fasting (h)	16.0 ± 6.9	15.9 ± 4.0	0.728^1^
Postoperative fasting (h)	39.2 (17.5-49.1)	40.9 (21.0-46.5)	0.175^2^
Perioperative fasting (h)	57.9 ± 20.5	59.7 ± 22.3	0.917^1^
	**LOS after surgery < 6 days (n = 35)**	**LOS after surgery ≥ 6 days (n = 42)**	**P**
Preoperative fasting (h)	17.4 ± 6.1	14.7 ± 4.3	0.001^1^
Postoperative fasting (h)	36.3 (16.5-41.3)	45.0 (33.4-69.8)	0.001^2^
Perioperative fasting (h)	50.4 ± 14.5	66.1 ± 23.8	< 0.001^1^

1 Student t test (data presented as mean ± standard deviation);

2 Mann-Whitney test [data presented as median (P25-P75)].

Logistic regression was performed to evaluate the association between prolonged hospitalization after surgery (≥ 6 days) and the duration of perioperative or postoperative fasting (Table 4). For each one-hour increase in the duration of perioperative fasting, the odds of prolonged hospitalization increased by 12%; while for each one-hour increase in the duration of postoperative fasting, it increased by 5%. In the multivariate analysis adjusted for confounders, the duration of perioperative or postoperative fasting was not an independent predictor of postoperative complications or death in the hospital. A one-hour increase in the durations of postoperative and perioperative fasting increased the odds of death by 14% and 15%, respectively, but it did not reach statistical significance after adjustment for confounders.

**Table 4:** Association between fasting and clinical outcomes, from multivariate analyses

	Mortality OR (95% CI)	P
Postoperative fasting (h)^1^	1.04 (0.99-1.09)	0.069
Postoperative fasting (h)^2^	1.14 (0.99-1.31)	0.065
Perioperative fasting (h)^1^	1.05 (1.00-1.10)	0.044
Perioperative fasting (h)^2^	1.15 (0.99-1.34)	0.071
	**Postoperative complications OR (95% CI)**	**P**
Postoperative fasting (h)^1^	1.00 (0.96-1.05)	0.967
Postoperative fasting (h)^3^	0.96 (0.92-1.01)	0.099
	**Prolonged hospitalization (length of hospital stay after surgery > 6 days) OR (95% CI)**	**P**
Postoperative fasting (h)^1^	1.05 (1.02-1.07)	0.001
Postoperative fasting (h)^4^	1.01 (1.04-1.17)	0.002
Perioperative fasting (h)^1^	1.05 (1.02-1.08)	0.002
Perioperative fasting (h)^4^	1.12 (1.04-1.20)	0.002

Logistic regression. OR = odds ratio; 95% CI = 95% confidence interval.

1 Crude model;

2 model adjusted for age, nutritional status according to PG-SGA, tumor site and occurrence of complications in postoperative period;

3 model adjusted for age, nutritional status according to PG-SGA, tumor site, tumor stage and length of postoperative hospitalization;

4 model adjusted for age, nutritional status according to PG-SGA, tumor site, tumor stage and occurrence of complications in postoperative period.

## DISCUSSION

The aim of the current study was to evaluate the duration of perioperative fasting and its association with postoperative complications, LOS and mortality in a sample of surgical patients with gastric and colorectal cancer. The mean duration of perioperative fasting was long (59 hours). Although fasting was not associated with the incidence of postoperative complications and death, a one-hour increase in perioperative fasting was associated with an increase of 12% in the odds of prolonged hospitalization after surgery. To date, no other study on cancer patients has evaluated the impact of perioperative fasting on selected outcomes (postoperative complications, LOS and death).

Nutritional care for cancer patients undergoing surgery extends well beyond the perioperative period. The adverse effects of prolonged fasting on glucose metabolism have already been reported,^16^^–^^18^ but the effects relating to postoperative complications remain unclear. Conventionally, feeding for patients undergoing gastrointestinal surgeries has been prescribed only after the return of peristalsis, clinically characterized by the appearance of bowel sounds and elimination of flatus.^19^ However, nowadays, experts are contesting this type of conduct, because evidence has shown that early feeding can be administered with minimal risks and with potential benefits for patients.^20^ The same has been described in relation to preoperative fasting, and there is a consensus in the literature regarding the importance of keeping this short. This is extremely important in populations such as that of the present study, with high frequency of diabetes (22%) and overweight (54.6%), as these individuals present the characteristics of oxidative stress and inflammation.

The mean duration of preoperative fasting was approximately 16 hours. This was longer than recommended, even in conservative prescriptions, but was in line with what had been reported in other studies without any implementation of measures to shorten the fasting protocol. Pereira et al. observed that the average duration of preoperative fasting in a sample of 128 surgical cancer patients was 26.4 ± 47.1 hours, but the average total length of fasting was 107.6 ± 73 hours.^21^ Falconer et al. interviewed 292 surgical patients, of whom 192 (65.8%) had undergone elective operations. All of the elective patients had received instructions for preoperative fasting of duration longer than six hours.^20^ The results showed that the mean duration of preoperative fasting from solids was 13.5 hours (interquartile range, IQR 11.5-16 hours). A variety of clinical factors may require prolonged preoperative fasting, such as for patients with a high risk of aspiration and those with medical conditions that may delay gastric emptying.^20^ Aguilar-Nascimento et al. believed that both clinicians and patients might still believe that fasting from midnight onwards was safer.^22^

Although the duration of perioperative fasting was considered high, it was not associated with complications in the postoperative period. To the best of our knowledge, this study was the first to describe this relationship, and probably the prolonged fasting was not due to the patients’ conditions, but to those of the healthcare establishment, such as the availability of the surgical center and the medical staff. Despite preoperative nutritional care, patients remain at risk of postoperative complications and deterioration of nutritional status.^7^

The results from the present study also show that non-survivor patients experienced a more prolonged duration of post and perioperative fasting. In the multivariate analysis, the durations of postoperative and perioperative fasting were probably not independent factors for mortality due to a lack of study power, given that the incidence of death was low in the current study. There is also a dearth of prospective studies about perioperative fasting focusing on selected outcomes (complications and mortality) among patients undergoing major surgery for cancer.

Receiving nothing orally for a long time preoperatively constitutes a persistent intervention and results in discomfort among patients. Clinical protocols should therefore be revised.^23^ In a case study,^24^ three patients were followed before and after laparoscopic colorectal resection: one with control fasting, another with shortened preoperative fasting and the third with shortened pre and postoperative fasting. Even with the shortened protocol, the latter two patients underwent preoperative fasting for approximately 17 hours, while the control patient remained in a fasting state for 43 hours. Additionally, the control patient showed increasing preoperative discomfort (hunger, thirst and anxiety), compared with the other two patients. However, the mean duration of hospital stay was not statistically significant different.^24^

Another interesting finding from the present study was that there was an association between the duration of perioperative fasting and prolonged hospitalization after surgery. A one-hour increase in the duration of perioperative fasting increased the odds of prolonged hospitalization by 12%; while a one-hour increase in the duration of postoperative fasting increased the odds of this outcome by 5%. An interesting study described the duration of preoperative fasting at a single center among surgical cancer patients before and after implementation of the enhanced recovery after surgery (ERAS) protocol.^19^ With the implementation of this protocol, the authors observed a significant decrease in the duration of preoperative fasting (14.7 [4-48] hours versus 7.2 [1-48] hours), but without any difference in the length of postoperative hospital stay (3.9 [0-51] versus 3.2 [0-15] days; P = 0.52). This was similar to the results from the present study, i.e. among patients whose duration of preoperative fasting was up to five hours, the length of hospitalization decreased by one day (3.8 [0-51] versus 2.5 [0-15] days). Our results show the importance of reducing the durations of both pre and postoperative fasting.

Although several studies in the literature have shown that implementation of a shortening of the fasting protocol in surgical cases entailed shorter hospitalization and improved postoperative recovery, data from cancer patients are scarce. Pereira et al. evaluated 128 patients who underwent surgical treatment for gastrointestinal cancer.^21^ The total length of fasting (mean of 107.6 hours) was significantly associated with the number of symptoms presented before and after the surgery. Differently from the present study, those authors did not evaluate surgical complications in accordance with the Clavien-Dindo scale. In a systematic review on shortening of fasting among patients undergoing oncological surgery, Pinto et al. analyzed four studies with a total of 150 patients (128 with colorectal cancer and 22 with gastric cancer).^25^ In comparison with traditional protocols, patients undergoing shortening of fasting through administration of fluids containing carbohydrates showed improvements in glycemic and inflammatory parameters and in malnutrition indicators, along with shorter hospital stay.

This study has several limitations. First, the data collection method may have been a source of information bias since there is an element of subjectivity inherent in patient recall. It was also not possible to identify whether the fasting related only to medical prescription or whether the patients were unable to tolerate oral food, which probably related to the length of hospital stay. Second, the study was conducted in a single center and included patients with different cancer sites (heterogeneity of the study subjects and surgical procedures used). However, the baseline characteristics were not significantly different between the groups. Furthermore, the incidence of death in this sample was low, and it prevented us from performing multivariate analysis adjusted for confounders, in order to investigate the real association between perioperative fasting and mortality. In addition, the study was conducted using a convenience sample and the analysis should be considered exploratory since the power of the study to test the hypothesis was not predefined. This matter needs to be better explored in further studies with larger samples.

## CONCLUSION

Prolonged perioperative fasting was observed in this sample of patients with gastric and colorectal cancer and it was an independent predictor of the length of hospital stay. This result emphasizes that there is a need for protocols to shorten fasting in this group of patients.
